# Eco-Friendly Bio-Hydrogels Based on Antheraea Pernyi Silk Gland Protein for Cell and Drug Delivery

**DOI:** 10.3390/gels8070398

**Published:** 2022-06-23

**Authors:** Jia Li, Bo-Xiang Wang, De-Hong Cheng, Yan-Hua Lu

**Affiliations:** 1 School of Chemical Engineering, Liaodong University, Dandong 118003, China; lj18840597623@163.com (J.L.); bxwang0411@163.com (B.-X.W.); 2 Liaoning Provincial Key Laboratory of Functional Textile Materials, Liaodong University, Dandong 118000, China

**Keywords:** Antheraea Pernyi, silk gland protein, sodium alginate, RSC96 cell, drug loaded bio-hydrogels, tissue engineering

## Abstract

The Antheraea Pernyi silk gland protein originates from natural organisms and synthesized by tussah silk glands and has widely potential biomaterial applications due to the superior biocompatibility. This study investigates the Antheraea Pernyi silk gland protein-based drug-loaded bio-hydrogels for bioengineered tissue fabricated by using an eco-friendly method without the harsh extracting process and the usage of toxic chemicals. The drug-loaded bio-hydrogels exhibited a porous structure and interconnected pore walls. The swelling ratio and water absorption of drug-loaded bio-hydrogels were, respectively, above 95% and 1.5 × 10^3^%. The cumulative release of drug loaded hydrogels all reached more than 90% within 4 h, and this indicates the potential of drug-loaded hydrogels as future drug-carrying biomaterials. RSC96 Schwann cells cultured on drug-loaded hydrogels for 72 h under cell culture medium show no toxic effects and more pro-proliferative effects. The results suggest the suitability of drug-loaded bio-hydrogels as natural biopolymer for the potential in vitro RSC96 cell culture platform and other biomaterial applications.

## 1. Introduction

Tissue engineering is an advanced technology for treating the failure of tissues and organs with new tissues grown in vitro [1,2,3]. The key to tissue regeneration is biocompatible scaffolds that can provide mechanical support for cell growth and format new tissues [4]. Hydrogels have become one of the most promising materials in the field of polymeric materials for the formation of biocompatible scaffolds [5]. They can simulate extracellular matrix and create environment suitable for faster cell growth in vitro, since they have the high water content, good flexibility, and good biocompatibility and also provide mechanical support for cell growth and format new tissue [6,7]. It is crucial to choose the appropriate materials for ensuring the effectively functions of hydrogels. Among the naturally polymers for biological applications, silk protein, and sodium alginate are widely explored for regenerative medicine due to biocompatibility, biodegradation, and non-toxicity to the human body [8,9,10].

Sodium alginate is a kind of rich natural polysaccharide, which is composed of β -D-mannouronic acid (M unit) and α -L-guronuronic acid (G unit) linked by 1, 4-glycosidic bond to form random block copolymer [11]. However, the weak mechanical properties, high brittleness, poor biodegradability, and bad biocompatibility of sodium alginate material have limited the application range of sodium alginate. In order to broaden the application range of sodium alginate in biomaterials, it would be effective for the modification of sodium alginate biomaterials to be introduced to a multi-level network structure.

Protein is derived from organisms and has no physiological toxicity. As one of the best sources of polymer materials, they are an important research object [12]. They can be introduced into the sodium alginate multi-level network as a natural biomaterial to construct the natural composite biomaterial of sodium alginate [13,14,15]. As a biocompatible material, silk protein of Antheraea Pernyi is a unique wild protein in China. Antheraea Pernyi silk protein originates from natural organisms and is synthesized by tussah silk glands [16]. Silk protein is widely used in biotechnology and biomedical fields due to the characteristics of high strength, blood compatibility, high permeability to water and oxygen, and interaction with mammalian cells [17]. Tussah silk protein has a unique natural arginine–glycine–aspartate RGD tripetide sequence, which can produce specific interactions with mammalian cells and improve cell adhesion and growth [18,19,20]. Degradation products of tussah silk protein are polypeptides and free amino acids with the characteristic of non-toxic to human body. They are widely used in biotechnology and biomedical areas such as antioxidants, anticoagulants, and wound healing due to their better biological affinity and cell adhesion than other biological proteins [21,22,23]. However, silk protein structure is damaged, and molecular weight is low when the traditional regenerative silk protein extraction process is used. In order to further apply the silk protein, it is often necessary to modify, graft, and blend with other materials [24,25].

Although the above studies have improved the mechanical strength and biodegradability of regenerated proteins or endowed them with special functions, the regenerated proteins are only biological proteins in concept, not “living” biological proteins in a real sense. Therefore, tussah silk gland protein obtained from tussah silk glands is a real “live” original ecological biological. It not only fixes the regenerated proteins’ small molecular weight and the poor mechanical properties but also improves the mechanical properties and endows hydrogels with new biological “live” sodium alginate.

In this study, we have investigated the possibility of using Antheraea Pernyi silk gland protein in its natural state for the fabrication of Antheraea Pernyi silk gland protein/sodium alginate drug-loaded hydrogels endowed with new biological “live” properties to be used as bio-based material. This method not only excludes the structural destruction of silk protein during the harsh extracting process but also avoids usage of toxic chemicals for extraction compared to conventional LiBr method. This study intends to promote the usage of native undegraded Antheraea Pernyi silk gland protein for the preparation of drug loaded bio-hydrogels for various biotechnological and tissue engineering applications.

## 2. Results and Discussion

### 2.1. ASGP Characterizations

#### 2.1.1. Amino Acid Composition and Content Analysis

Amino acid composition and content determination of adenin of tussah silk are shown in Table 1. Amino acid composition analysis showed that there were about 18 amino acids in the ASGP, with plenty of alanine (Ala), glycine (Gly), tyrosine (Tyr), and serine (Ser), accounting for a total of 64.18%, among which serine accounts for 11.27%, glycine 20.07%, alanine 20.12%, and tyrosine 12.72%. The content of tyrosine was much higher than that of other aromatic residues phenylalanine (1.75%). Hydrophobic amino acids (Ala, Val, Leu, Ile, Pro, Phe, and Trpand Met), alkaline amino acids (Lys, ArgandHis), and acid amino acids (Asp, Glu) accounted for about 44.27%, 6.39%, and 12.07%, respectively. The amino acid composition of ASGP resembles that of traditional tussah silk fibroin [26,27]. Thus, the main component of tussah silk gland protein extracted in this study is silk fibroin.

#### 2.1.2. SDS-PAGE and CD Analysis

**Figure 1:**
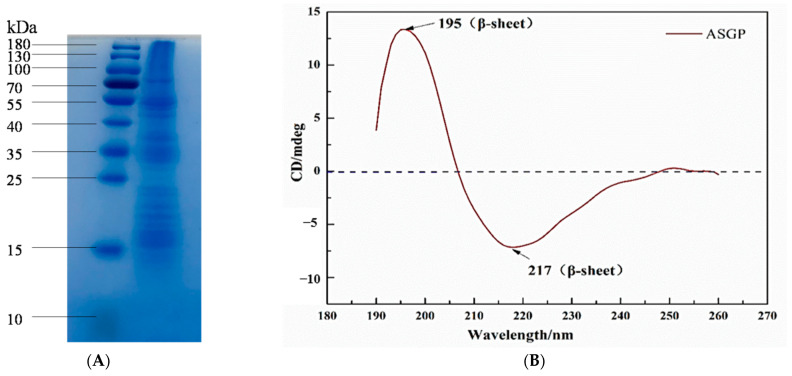
(**A**) SDS-PAGE and (**B**) CD analysis of ASGP.

**Figure 2:**
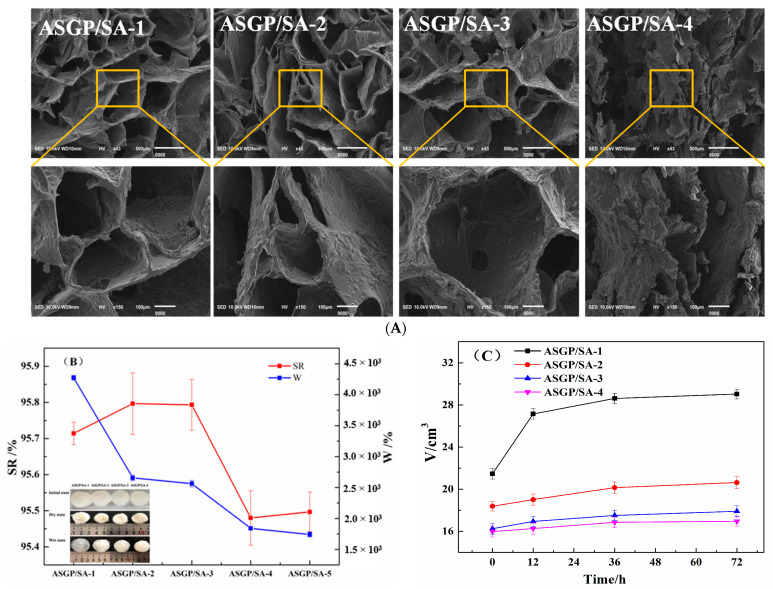
(**A**) SEM; (**B**) the swelling ratio (SR), water absorption (W), and the initial/Dry/Wet; states and (**C**) stability of drug loaded hydrogels.

SDS-PAGE was carried out to analyze the molecular weight of ASGP. The results are shown in Figure 1A. It can be seen from Figure 2A that ASGP has a continuous spectral band between 15 and 180 kDa and different molecular weights peptides. The results indicate that ASGP is a mixed polypeptide with a wide and relatively dense molecular weight distribution.

Circular dichroism (CD) was introduced to determine the secondary structure of ASGP (Figure 2B). In the far UV region of the CD spectrogram (185–240 nm), the secondary structure of protein (random curl, α-helix, β-fold and β-rotation) has characteristic absorption peaks. The characteristic peaks of α-helix structure was about at 192 nm (+), 208 nm (−), and 222 nm (−), and that of the β-sheet structure was at 195 nm (+) and 216 nm (−). The β-turn structure was known to have a weak characteristic absorption peak at 220–230 nm (−) and strong characteristic absorption peaks at 180–190 nm (−) and 205 nm (+). The characteristic absorption peaks of the random coil structure were at 200 nm (−) and 212 nm (+) [28]. The result of the secondary structure of ASGP is shown in the figure. It can be seen from the figure that a significant positive peak at 195 nm (+) and a significant negative peak at 217 nm (−) could be contributing to the β-sheet structure of ASGP. Thus, the main secondary structure of ASGP directly extracted from tussah silk gland was a β-sheet structure.

### 2.2. Morphological Observation

SEM was adopted to show the surface morphology of drug-loaded hydrogels. The SEM images of the samples exhibited a porous structure, and the apertures decreased with the increase in ASGP (Figure 2). The swelling and water absorption behavior of the support material reflects the ability of the solution to penetrate into the matrix and the stability of the matrix. Due to ionization, the concentration of ions in the hydrogel network is increased, resulting in osmotic pressure inside and outside of the hydrogel, which is balanced by the swelling of the hydrogel. The swelling of the loaded gel directly affects the drug release performance [29]. The swelling ratio and water absorption of samples is given in Figure 2B. It can be found from Figure 2B that the swelling ratio and water absorption are, respectively, above 95% and 1.5 × 10^3^%. With the increasing content of ASGP, the swelling and water absorption of hydrogel decrease gradually. This could be because the CA strengthened the intermolecular interaction between the SA chains. This is also because the reduction in cross-linking point and the loosening of the structure serve to accelerate the release of drugs with the decreasing content of sodium alginate [30]. The stability of drug-loaded hydrogels is given in Figure 2C. It can be seen that all the samples can keep the original hydrogels state and that the volume showed no obvious change in the SIF for 72 h. The results shows that citric acid and calcium chloride can be used as a medicine gel composite curing agent for preparing a stable drug-loading hydrogel.

### 2.3. FT-IR Analysis

**Figure 3:**
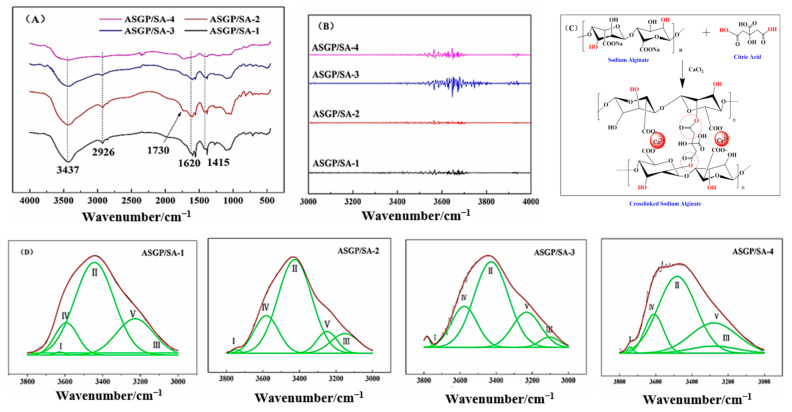
(**A**) FT-IR spectra, (**B**) the second derivative spectra, (**C**) solidifying mechanism, and (**D**) Gauss fitting curves of drug-loaded hydrogels.

The drug-loaded hydrogels were characterized by FT-IR (Figure 3A). According to the FT-IR results, the absorption peaks of 3437 cm^−1^, 2926 cm^−1^, 1620 cm^−1^, and 1415 cm^−1^ were related, respectively, to -OH the stretching vibration, -CH of six-membered ring stretching vibration, -COO- asymmetric stretching vibration, and symmetric stretching vibration [31]. The FT-IR spectra of hydrogels was shown in Figure 3A. The figure shows the absorption peak at 3437 cm^−1^ for stretching vibration of -OH; 2926 cm^−1^ for six-membered ring stretching vibration of -CH; 1730 cm^−1^ for carbonyl bands (carboxyl and ester), confirming the curing formation (see Figure 3C); 1620 cm^−1^ for asymmetric stretching vibration of -COO-; and 1415 cm^−1^ for symmetric stretching vibration of -COO-. They belong to SA and ASGP, respectively.

The infrared spectroscopy of drug loaded hydrogels was smoothed using the Savitzky–Golay method and treated with the Gauss peak fitting of peak analyzer and second-derivative spectra for studying the hydrogen bond interaction among SA, ASGP, and curing agents ranging from 3000 to 3800 cm^−1^ (Figure 3B,D). The hydrogen bonds of different types of SA, ASGP, and curing agents are shown in Figure 3D and Table 2. Gauss fitting results in Table 3 suggest that the intermolecular hydrogen bond content of SA and ASGP increased, respectively, from 18.68% to 26.46% and 38.82% to 39.02%, and the intramolecular hydrogen bond content of SA and ASGP decreased, respectively, from 80.05% to 72.95% and 60.16% to 60.28% with the increasing content of ASGP. The results showed that there is a strong interaction among SA, ASGP, CA, and CaCl_2_.

### 2.4. UV-Vis Analysis

**Figure 4:**
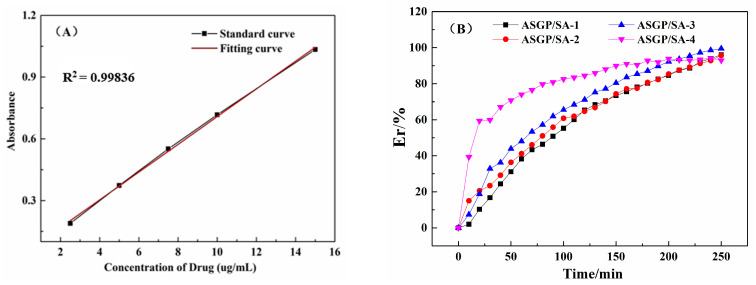
(**A**) Standard curve of MFH and (**B**) The drug-release performance of drug-loaded hydrogels.

The drug release performance of drug-loaded hydrogels is shown in Figure 4. As Figure 4A,B illustrates, all samples had drug-release activity. The cumulative drug releases of the drug-loaded hydrogels all reached more than 90% within 4 h. Additionally, the drug-release rate of samples gradually increased with the increase in ASGP content. On the one hand, this is attributed to the existence of large amounts of polar groups, such as hydroxyl, carboxyl, amino, and ether bonds, which can form hydrogen bonds with distilled water. With the increase in ASGP content, there will be more intermolecular hydrogen bonds formed, seeing intermolecular forces enhanced. This is also because the hydrogels were fully dilated after contacting the SIF, which provides enough free movement space for the silk-gland protein peptide chain [32]. It is conducive to the transformation of the silk-gland protein β-sheet into a random curl that is conducive to the drug release from the drug-loaded hydrogels. On the other hand, this is also attributed to the carboxyl deprotonation of SA. The electrostatic repulsion of the dissociated carboxyl ions promoted the chain extension of SA and the drug was diffused and released through osmotic pressure. Additionally, beyond that, calcium ions in the hydrogels exchange with sodium ions in the solution, the force of phosphate ions on calcium ions in the medium is greater than that of SA, and the chelation between calcium ions and phosphate ions occurs [33,34].

### 2.5. Cell Viability Assay

**Figure 5:**
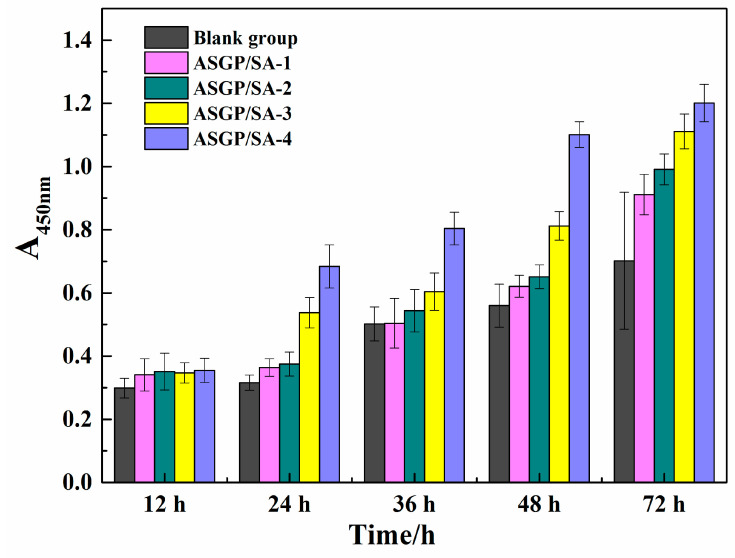
The cytotoxicity of drug loaded hydrogels.

CCK-8 assay was employed to investigate the biocompatibility of the drug-loaded hydrogels. The cells’ viability was investigated according to the incubation with the samples after 12, 24, 36, and 72 h (Figure 5). No toxic effects were observed in any samples. Compared with the drug-loaded hydrogels samples showed more pro-proliferative effects on the RSC96 Schwann cells, which became obvious upon 72 h of incubation. The results illustrated that it had no effect on the cells’ proliferation with the increase in ASGP and that the composite drug-loaded hydrogels system would be a promising material to treat serious gastrointestinal diseases.

## 3. Conclusions

This study provides a significant insight into drug-loaded bio-hydrogels based on Antheraea Pernyi silk gland protein cues playing a role in drug loading and cell proliferation. In this study using Antheraea Pernyi silk gland protein/sodium alginate drug-loaded hydrogels models were able to show a swelling ratio and water absorption above 95% and 1.5 × 10^3^%, more than above 90% cumulative drug release for 250 min, and more pro-proliferative effects on cells for 72 h due to the higher porosity and pore size. We further envisage that optimizing bio-hydrogels based on Antheraea Pernyi silk gland protein may safely be implemented as pharmaceutical carriers and as a cell culture plat form in the future to achieve enhanced cell support, proliferation, and differentiation of cells with the aim of developing efficient Antheraea Pernyi silk gland protein for other biomaterial applications.

## 4. Experimental Section

### 4.1. Materials

Fifth-instar larvae of Antheraea pernyi were collected from local farms (Dandong, Liaoning Province, China). Sodium alginate (SA, M*_W_* = 5.0 × 10^3^ KDa) was purchased from Qingdao Bright moon Seaweed Group (Qingdao, China). Metformin hydrochide (97%, MFH) was purchased from Shanghai Macklin Biochem Tech (Shanghai, China). The RSC96 cellline was purchased from Guangzhou JinioBio technology Co., Ltd. (Guangzhou, China). The CCK-8 Kit (Cell Counting Kit-8) was purchased from Biosharp White Shark Biotechnology Co., Ltd. (Shanghai, China). Cell culture medium (sterile filtration) was purchased from Ge Healthcare Life Sciences Hyclone Co., Ltd. (Beijing, China). Artificial blood was purchased from Dongguan Chuangfeng Technology Co., Ltd. (Guangdong, China). All other materials and reagents were purchased from Aladdin Bio-Chem Technology Co., Ltd., (Shanghai, China). All the reagents are AR.

### 4.2. Extraction of Antheraea Pernyi Silk Gland Protein

The fifth-instar larvae of Antheraea Pernyi were dissected, and the Antheraea Pernyi silk glands were obtained. The silk glands were cleaned with distilled water three times and refrigerated at −30 °C for later use. The silk glands above 1.0 g were solubilized with 1% Sodium Dodecyl Sulfate (SDS) in Tris•HCl solutions (0.01 M, pH = 8.0) under oscillation for about 2 h at room temperature. The resulting mixture was centrifuged at 8000 rpm, and the solubilized silk gland protein (ASGP) was obtained and refrigerated at 4 °C for later use.

### 4.3. Preparation of Drug Loaded Hydrogels

**Figure 6:**
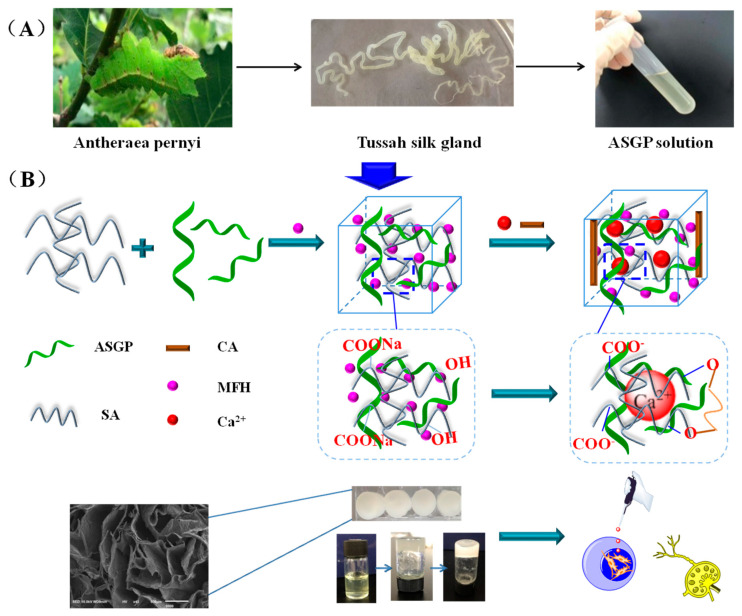
Schematic illustration of the (**A**) extraction of ASGP; (**B**) preparation of drug-loaded hydrogels.

The above solubilized silk gland proteins were added to 20 mL 5% sodium alginate solution and 15 mg MFH to obtain the drug carrier ASGP/SA mixed solution. The above mixed solutions were poured into a composite coagulation bath including 14% citric acid (CA) solution and 0.1% CaCl_2_ solution for about 12 h to obtain drug-loaded hydrogels. The illustration of the drug-loaded hydrogels is shown in Figure 6.

### 4.4. Testing and Analysis

#### 4.4.1. Determination of Amino Acid Composition

The amino acid composition of ASGP was determined by Biochrom Amino Acid Analyzer (Biochrom, Cambridge, UK) in the flow rate of 20 mL/h and a reaction flow rate of 10 mL/h. Na-type cationic resin chromatography column was used with the 200 mm long and 4.6 mm in diameter. The UV detection wavelengths were 570 nm and 440 nm. The programmed temperature of column, reaction tank temperature, and sample volume were 55–65–77 °C, 138 °C and 50 µL, respectively.

#### 4.4.2. SDS-PAGE Test

SDS-PAGE was carried out to estimate the molecular weight of ASGP. Samples weighing about 0.2 μg were loaded on a 12% stacking gel cast at the top of a 5% SDS polyacrylamide gel and then electrophoresed under the applied voltage of 80–100 V. After electrophoresis, the gel was stained with Coomassie Brilliant Blue solution (G250, 45% methanol, 10% acetic acid) for about 1 h and distained with solution including 5% methanol and 7.5% acetic acid in water.

#### 4.4.3. CD Assay

The secondary structure of ASGP was characterized by a MOS-450 circular dichromatic spectrometer. The sample solution concentration was 0.2 mg/mL. The power supply and the flow rate of nitrogen flow meter were 150 W and 7 L/min, respectively. It was stabilized for 20 min in N_2_ atmosphere and then locked. The absorbance mode was used at the scanning wavelength range from 180 to 260 nm, and the scanning time interval of 0.05 s.

#### 4.4.4. Swelling Ratio, Water Absorption and Stability Test

All the dried-drug-loaded hydrogels samples were taken out, and the top layer of gel scaffolds was removed and then dried at 40 °C for 4 h. The swelling ratio (SR) and the water absorption(W) were, respectively, volumetrically and gravimetrically monitored by immersing the dried hydrogels samples (V_1_, W_1_) in water for 24 h. The mass of swollen gel scaffolds (V_2_, W_2_) was measured after removing residual moisture. The stability of drug-loaded hydrogels samples was performed volumetrically by immersing the dried hydrogels samples in SIF for 0 h, 12 h, 36 h, and 72 h. The swelling ratio (SR) and the water absorption(W)were, respectively, calculated as follows (1), (2):

(1) $$\mathrm{SR}(\%)=\frac{V_{2}-V_{1}}{V_{1}}\times 100\%$$

(2) $$W(\%)=\frac{W_{2}-W_{1}}{W_{1}}\times 100\%$$

#### 4.4.5. SEM Assay

SEM (JSM-IT100, JEOL Ltd., Tokyo, Japan) was introduced to observe the dried-drug-loaded hydrogels samples after they were quenched in liquid nitrogen and sputtered with a thin layer of gold.

#### 4.4.6. FT-IR Assay

FT-IR spectroscopy (Nicolet IS10 FT-IR spectrometer, Thermo Fisher Scientific, Waltham, MA, USA) was employed to analyze the chemical structure of drug-loaded hydrogels. The pure sample powders were prepared with the KBr technique at the wavelength range of 400–4000 cm^−1^.The samples were obtained in a vacuum dryer for 4 h before the spectra were acquired.

#### 4.4.7. UV-Vis Assay

The content of MFH in the drug-loaded hydrogels was evaluated by UV-Vis spectrophotometer (UV-T600, Beijing General Instrument Co., Ltd., Beijing, China), with 230 nm as the test wavelength. The artificial intestinal simulation fluids (SIF, pH = 7.4) was prepared by 0.05 M KH_2_PO_4_ and 0.0395 M NaOH solution. To obtain a standard curve of MFH, the 50 mg MFH was carefully placed in a 100 mL volumetric flask with SIF. Additionally, then a certain amount of MFH standard solution was taken and diluted with SIF to obtain 2.5 μg/mL, 5.0 μg/mL, 7.5 μg/mL, 10 μg/mL, and 15 μg/mL solutions. SIF was used as blank contrast sample, and the absorbance of MFH standard solution with different concentrations above was measured with a UV-Vis spectrophotometer. The standard curve of MFH solution was drawn by using an MFH solution concentration as abscess and absorbance as ordinate.

Drug release rate is measured as follows: the drug-loaded hydrogels (150 mg) were placed in a 100 mL SIF under the condition of 37.0 ± 0.5 °C for magnetic stirring. Additionally, the 5 mL samples were then taken at the same interval (completed within 30 s), and the same amount of dissolution medium was added. The absorbance of the sample was measured at wavelength 230 nm with an ultraviolet spectrophotometer. Drug cumulative release was calculated as follows (3):

(3) $$E_{r}\left(\%\right)=\frac{C_{n}\cdot V+V_{i}\sum_{i=0}^{n-1}C_{i}}{M_{0}}\times 100$$

where C*_n_* is the concentration of the sample drug solution at the nth time point; V is the total volume of dissolved medium; V*_i_* is the sampling volume at the *i*th time point; C*_i_* is the concentration of sample drug solution at the *i*th time point; and both V_0_ and C_0_ are zero. M_0_ is the mass of the drug loaded into the drug-loading hydrogels. Three groups per sample were tested, and the average value was calculated.

#### 4.4.8. Cell Viability Assay

CCK-8 (cell counting kit-8) assay was employed to evaluate the biocompatibility of the drug-loaded hydrogel samples. The RSC96 cells suspension at the logarithmic growth stage were prepared with culture medium, serum, and dual antibody and cultured in a CO_2_ incubator (Thermo Fisher Scientific Instruments, Waltham, MA, USA), with 5% saturation at 37 °C for three days. A one hundred microliter RSC96 cell suspension was diluted and then transplanted and cultured on the cell plate for 24 h. The cell culture medium was sucked out and replaced with a new one. Then, the treated drug-loaded hydrogel samples’ extract was added into the above cell culture medium and cultured for 12 h, 24 h, 36 h, 48 h, and 72 h in the same environment. The cell culture medium was used in place of the extract and set as the blank group. The cell culture medium was sucked out at different times, and the same amount of CCK 8 medium mixture was added. The mixture was then placed into a CO_2_ incubator for 30 min. The optical density was recorded at 450 nm by a microplate reader. The test was performed in triplicate. Cell toxicity was determined by cell survival rate, and the cell survival rate was calculated according to Formula (4):

(4) $$C.V.=\frac{A_{\mathrm{sample}}}{A_{\mathrm{control}}}\times 100\%$$

where C.V., A_sample_, and A_control_ are cell survival rates, the OD value of sample extraction solution after cell culture, and the OD value of the solution in the blank group, respectively.

## Figures and Tables

**Table 1:** Amino acid composition of ASGP, SF and SS.

Amino Acids	ASGP	SF	SS
	Mole/%	Mole/%	Mole/%
Asp	8.87	5.11	14.86
Thr	1.18	0.44	15.16
Ser	11.27	11.03	19.94
Glu	3.20	0.90	7.07
Gly	20.07	29.81	8.69
Ala	20.12	42.27	2.14
Cys	0.91	0.23	0.27
Val	3.08	0.88	2.18
Met	3.45	0.00	0.12
Ile	3.00	0.41	1.50
Leu	1.83	0.41	1.91
Tyr	12.72	5.11	7.28
Phe	1.75	0.34	1.05
His	2.55	0.90	5.58
Lys	1.46	0.13	1.54
Try	2.17	1.75	0.20
Arg	2.38	2.72	6.29
Pro	8.87	0.00	3.33

**Table 2:** Specific preparation parameters of hydrogels.

Sample	ASGP/g	5%SA/mL	MFH/mg	0.1%CaCl_2_/14%CA(*v*/*v*)
ASGP/SA-1	0.2	16	15	5/5
ASGP/SA-2	0.4	12	15	5/5
ASGP/SA-3	0.6	8	15	5/5
ASGP/SA-4	0.8	4	15	5/5

**Table 3:** Gauss fitting results of hydrogen bond for drug-loaded hydrogels.

Sample	Hydrogen Bond Type	Abbreviations		Wavenumbers/cm^−1^	Relative Strength/%	
ASGP/SA-1	Free hydroxyl	Ⅰ	-OH	3634	1.27	1.27
	Intramolecular	Ⅱ	OH…OH	3438	76.69	80.05
	hydrogen bond	Ⅲ	Annular polymer	3104	3.36	
	Intermolecular	Ⅳ	OH...π	3585	7.23	18.68
	hydrogen bond	Ⅴ	OH…ether O	3221	11.45	
ASGP/SA-2	Free hydroxyl	Ⅰ	-OH	3747	0.61	0.61
	Intramolecular	Ⅱ	OH…OH	3425	62.39	72.95
	hydrogen bond	Ⅲ	Annular polymer	3151	10.56	
	Intermolecular	Ⅳ	OH...π	3581	17.93	26.44
	hydrogen bond	Ⅴ	OH…ether O	3249	8.51	
ASGP/SA-3	Free hydroxyl	Ⅰ	-OH	3784	1.02	1.02
	Intramolecular	Ⅱ	OH…OH	3427	56.41	60.16
	hydrogen bond	Ⅲ	Annular polymer	3103	3.75	
	Intermolecular	Ⅳ	OH...π	3540	20.02	38.82
	hydrogen bond	Ⅴ	OH…ether O	3231	18.80	
ASGP/SA-4	Free hydroxyl	Ⅰ	-OH	3741	0.70	0.70
	Intramolecular	Ⅱ	OH…OH	3481	54.30	60.28
	hydrogen bond	Ⅲ	Annular polymer	3209	5.98	
	Intermolecular	Ⅳ	OH...π	3609	13.48	39.02
	hydrogen bond	Ⅴ	OH…ether O	3279	25.54	

## Data Availability

Data are contained within the article.
